# Patterns of love and sexting in teen dating relationships: The moderating role of conflicts

**DOI:** 10.1002/cad.20427

**Published:** 2021-06-09

**Authors:** Dora Bianchi, Mara Morelli, Roberto Baiocco, Elena Cattelino, Antonio Chirumbolo

**Affiliations:** ^1^ Department of Social & Developmental Psychology Sapienza University of Rome Rome Italy; ^2^ Department of Dynamic and Clinical Psychology, and Health Studies Sapienza University of Rome Rome Italy; ^3^ Department of Human and Social Science University of Valle d'Aosta Aosta Italy; ^4^ Department of Psychology Sapienza University of Rome Rome Italy

**Keywords:** adolescents, commitment, conflicts, dating relationships, intimacy, passion, sexting

## Abstract

According to the triangular love theory, this study investigated the roles of three components of love (i.e., passion, intimacy, commitment) and the moderating role of conflicts in predicting different forms of sexting (i.e., experimental, nonconsensual, under pressure) in teen dating relationships. Participants were 409 adolescents (*M_age_* = 17.20, *SD_age_* = 1.61; 62.6% girls) who completed an online questionnaire. Three moderated regressions were performed. Conflicts positively predicted all forms of sexting. Passion positively predicted experimental sexting. Intimacy negatively predicted experimental and nonconsensual sexting, and positively predicted sexting under pressure. Three interaction effects emerged, pointing out the moderating role of conflicts. Passion positively predicted nonconsensual sexting in the presence of high conflicts, while this relationship became negative when conflicts were low. Commitment negatively predicted nonconsensual sexting and sexting under pressure in the presence of high conflicts, but these relationships were not significant when conflicts were low. Research and applicative implications are discussed.

## 1 INTRODUCTION

Sexting among adolescents has been a widely studied topic in the recent years, particularly due to its relevant risks for young people. It is widely acknowledged that teen sexting predominantly occurs in the context of a dating relationship (for a review see Cooper et al., 2016; Klettke et al., 2014). However, when the online sexual contents get out of control, or when they are misused to harass someone, all people involved in the sexting incidents may suffer negative consequences, including legal implications as well as adverse effects on psychological, emotional, and relational well‐being (Wolak & Finkelhor, 2011). Despite this awareness, the absence of different measurements for experimental (consensual and reciprocal) and aggravated (coercive and nonconsensual) forms of sexting is an important shortcoming in the current literature. Most studies applied indeed a general score of sexting behaviors, without differentiating between benevolent and aggressive intentions towards the partner (Mori et al., 2019). Other researches are specifically focused on some forms of aggravated sexting (review by Madigan et al., 2018; see also Morelli et al., 2016a; Reed et al., 2016), while neglecting the simultaneous evaluation of non‐aggressive sexting behaviors. Therefore, the specific characteristics of dating relationships which may favor experimental rather than aggravated forms of sexting are still unclear.

Recently, some authors have claimed the need to interpret sexting behaviors within the specific relational context in which they take place (Van Ouytsel et al., 2018, 2020), with the purpose of better understanding their associations with positive and negative relational correlates. Therefore, the present study aimed to fill this gap in literature, studying which components of love (i.e., passion, intimacy, and commitment) can predict experimental and aggravated sexting behaviors among teen dating partners within the theoretical framework of the triangular theory of love (Sternberg, 1997). In line with recent research (Van Ouytsel et al., 2019a), we also assessed the perception of conflicts with one's partner, in order to understand whether specific combinations of conflicts and love components characterize the relational contexts in which aggravated or experimental sexting may arise.

### 1.1 Sexting in teen dating relationships

Sexting consists of self‐made sexual contents, such as sexually suggestive text messages, images, or videos, which are shared via Smartphone applications, Internet, and social networks (Chalfen, 2009). This practice mainly occurs in the context of a romantic relationship, as a new kind of sexual and intimate communication (Cooper et al., 2016; Lenhart, 2009). Prevalence of sexting ranges between 14% and 48% among adolescent dating partners, with percentages varying according to the definitions adopted in different studies (Bianchi et al., 2019; Burén & Lunde, 2018). Adolescent sexting tends to increase with age and with pubertal development (Bianchi et al., 2019b; Burén & Lunde, 2018; Cooper et al., 2016), following the normal course of other sexual behaviors. Findings on gender differences are mixed: while it is acknowledged that, in the context of a dating relationship, sexting does not differ by gender or biological sex (Cooper et al., 2016; Klettke et al., 2014), other findings suggest, however, that boys tend to engage more frequently in risky sexting behaviors and to perpetrate more aggravated sexting (Morelli et al., 2020) than girls. Girls instead are more frequently victims of nonconsensual sexting which has also been considered a form of gender‐based violence (review by Krieger, 2017). On the other hand, studies consistently indicated that sexual minority adolescents engage in sexting more often than their heterosexual peers (Morelli et al., 2016b, 2020; Rice et al., 2014), also in the context of romantic relationships (Van Ouytsel et al., 2018).

The extant research on sexting is predominantly based on samples from the United States. (Cooper et al., 2016; Klettke et al., 2014) or Northern and Middle European countries (e.g., Gámez‐Guadix et al., 2017; Kopecký & Szotkowski, 2018; Van Ouytsel et al., 2017, 2018), but the present is one of the few studies conducted among Italian adolescents. According to the very limited evidence from Italy, 63% of Italian young people admit to send sexts, but only 47% report to send images or videos depicting themselves (Morelli et al., 2016b, 2020). Moreover, 3.3% of Italian youth have been forced into sexting by a dating partner, while around 12% have forwarded the partner's sexts without his/her consent (Morelli et al., 2016b). Finally, Italian adolescents who engage in sexting are predominantly moved by experimental motivations (80%), whereas only a minor percentage (14%) is moved by aggressive intentions (e.g., to harass or embarrass someone; Bianchi et al., 2018).

Past research has shown that sexting in teen dating relationships is predominantly consensual and voluntary, constituting an expression of sexual and romantic interest (Burén & Lunde, 2018; Lenhart, 2009). These behaviors, defined “experimental” (Wolak & Finkelhor, 2011), represent expressions of developmental instances, such as exploration of sexuality, intimacy, and identity (Levine, 2013). Also, experimental sexting is the most frequent form of sexting among young people, constituting between 56% and 88% of all sexting behaviors (Bianchi et al., 2016, 2018). Motivations underpinning experimental sexting among adolescent dating partners are related to flirting, joking, and showing sexual interest in the initial phase of a relationship, or enhancing intimacy and passion in established relationships (Cooper et al., 2016; Lenhart, 2009).

However, besides these experimental behaviors, also more aggressive forms of sexting may occur. These forms are defined “aggravated” since the sexual contents are misused for hurting or damaging the partner, implying sexual victimization or exploitation (Wolak et al., 2012). The most studied kinds of aggravated sexting are coercive and nonconsensual sexting. Sexting coercion consists of pressuring, threatening, or forcing the partner to engage in sexting, and the prevalence of this phenomenon in teen dating relationships is around 12% for victimization, and 8% for perpetration (Kernsmith et al., 2018). Nonconsensual sexting instead is an indirect aggression, consisting in forwarding sexts of the partner to other people without his/her consent, often with severe negative consequences for all minors involved. The prevalence of nonconsensual sexting is estimated around 12% among adolescents (Madigan et al., 2018).

Aggravated and experimental sexting highly differ in terms of emotional and psychosocial correlates (Bianchi et al., 2018; Klettke et al., 2014; Van Ouytsel et al., 2020), even if there are still a few studies which simultaneously measure and distinguish the two behaviors. Therefore, recent research has shed light on the importance to study sexting within the specific relational context in which it occurs (Van Ouytsel et al., 2018, 2020). Experimental and aggravated sexting behaviors might have indeed a very different impact on dating relationship adjustment and well‐being.

Literature on the impact of sexting on dyadic adjustment is still quite limited to adult samples, with various and contrasting findings (Currin et al., 2016; Galovan et al., 2018; Parker et al., 2013). In these studies, sexting within a romantic relationship has been linked to more sexual and dyadic satisfaction (Galovan et al., 2018; McDaniel & Drouin, 2015; Parker et al., 2013), but also to high conflict and ambivalence, low commitment, and low attachment security (Galovan et al., 2018), and more anxious and avoidant attachment styles (Drouin & Landgraff, 2012).

Conversely, very little is known about the role of sexting in relationship adjustment among adolescents (Reed et al., 2020; Van Ouytsel et al., 2019a). Teen sexting with a dating partner has been found positively associated with conflict and passion (Van Ouytsel et al., 2019a), and with anxious and avoidant attachment styles (Reed et al., 2020). These few studies are also very limited in that they do not distinguish between experimental and aggravated sexting, which however might show very different associations with relationship adjustment. Previous literature has demonstrated indeed that aggravated sexting—both coercive and nonconsensual—often occurs in a broader context of dating violence (Morelli et al., 2016a, 2017; Reed et al., 2016), and in association with offline aggression (Kernsmith et al., 2018), while experimental sexting is unrelated to aggressive dynamics (Bianchi et al., 2018).

### 1.2 Triangular theory of love in teen dating relationships

Adolescent romantic relationships can be studied within the theoretical framework of the triangular theory of love (Sternberg, 1997). This model conceptualized the presence of three components of love, that is, passion, intimacy, and commitment, which are present at different levels in couple relationships. Passion is characterized by arousal driven by sexual and romantic desires, and involves the need for physical proximity to the partner; intimacy refers to feelings of mutual trust, connectedness, and emotional closeness, and derives from the emotional investment in the partner; commitment is related to cognitive decisions to care for the partner and maintain the relationship over time (Deverich, 2009; Sternberg, 1997). Balanced levels of passion, intimacy, and commitment are supposed to characterize healthy relationships, so that the three components positively predict relationship satisfaction in adolescence as well as in young adulthood (Madey & Rodgers, 2009; Overbeek et al., 2007).

However, the three components of love can be present in different degrees during adolescence, influenced by the emotional, relational, and cognitive development (Deverich, 2009; Sumter et al., 2013). Passion is the strongest component in teen romantic relationships. Arising earlier in the relationship timing and progressively increasing with the pubertal growth, passion can reach a more complete expression than the other components during teenage (Deverich, 2009; Sumter et al., 2013). Intimacy in adolescent dating is mostly characterized by emotional disclosure, but it is limited by the incomplete definition in individual identity that during adolescence is in an explorative stage (Erikson, 1980). Adolescents who are still unsecure about themselves may not feel ready to merge their forming identities with a partner in a complete intimacy (Erikson, 1980), or conversely may tend to define themselves exclusively in the context of the relationship with the partner (Duvall, 1964). However, in these conditions, an intimate and secure relationship cannot develop, while insecure adolescents can escape from intimacy, or can use the relationship to escape own perceived dissatisfactions (Duvall, 1964; Erikson, 1980). Commitment in teen dating relationships is not comparable to commitment in adult couple relationships, due to the incomplete development of the prefrontal cortex which regulates the cognitive decisions to invest in and maintain the relationship in a long‐term perspective (Deverich, 2009). Conversely, the investment in the dating relationship during adolescence is more determined by emotional impulses and by peer influences, which have a stronger impact on adolescent decision making (Casey et al., 2008). Thus, commitment is more fleeting in adolescent relationships.

Studies on teen dating relationships have also revealed that the three components of love can lead to both positive and negative relational outcomes during adolescence. On one side, passion, intimacy, and commitment are positively linked to relationship satisfaction and duration; on the other side, high intimacy and commitment are also associated with more conflicts, and high passion with more perceived jealousy (Overbeek et al., 2007). Moreover, the construct of intimacy in teen dating relationships has been considered a combination of positive (e.g., trust and emotional closeness) and negative aspects (e.g., jealousy) (Matson et al., 2021). Finally, recent studies on teen dating violence have demonstrated that high trust and jealously, and low commitment are predictive of victimization and perpetration of dating aggression (Matson et al., 2021).

Only one study until today has suggested the possibility to predict adolescent sexting behaviors from the three components of triangular love (Van Ouytsel et al., 2019a), but the results are not very informative, indicating that sexting behaviors were positively related only to passion, while no significant associations emerged for intimacy and commitment. This unique research is also limited in that experimental and aggravated sexting behaviors were not distinguished from each other. Nevertheless, there is evidence that the components of love may lead to relationship well‐being (Overbeek et al., 2007), as well as dating aggression (Matson et al., 2021), suggesting that we should further observe the three components in their independent relationships with experimental and aggravated sexting.

Moreover, a meta‐analysis study (Graham, 2011) has demonstrated that the three components of the triangular love are not exhaustive of all aspects of love. If passion, intimacy, and commitment are useful to assess a general positive facet of love, there are also negative aspects related to dependent, possessive, and obsessive love, which are not captured by this model, and that negatively predict relationship satisfaction and well‐being (Graham, 2011).

Therefore, these aspects might be better understood when also the degree of conflict perceived in the relationship is taken into account, as suggested by previous research (Van Ouytsel et al., 2019a). Conflicts between intimate partners, such as frequency of arguing, have been often investigated in research since this measure allows to observe the conflictual relational climate beyond more specific and severe forms of aggression (Connolly et al., 2010; Connolly et al., 2010; Van Ouytsel et al., 2019a). Relationship conflicts have been indicated as a common feature in teen dating aggression (Adelman & Kil, 2007; Connolly et al., 2010), and high levels of verbal conflicts are predictive of different forms of dating violence (Katz & Myhr, 2008; O'Leary & Slep, 2003). Therefore, it is possible to hypothesize that a conflicting climate could be a positive predictor of aggravated facets of sexting behaviors, while the triangular love dimensions might be expected to predict experimental sexting. Moreover, relationship conflicts might be a moderator which could help to explain and contextualize the associations of passion, intimacy, and commitment with different kinds of sexting.

## 2 THE CURRENT STUDY

This study investigates the role of three components of love (passion, intimacy, and commitment) and of perceived dyadic conflicts in predicting different sexting behaviors (experimental, nonconsensual, and under pressure) in a sample of Italian adolescents who have, or have had, a dating relationship. Individual differences related to biological sex, age, and sexual orientation have been controlled for, on the basis of previous literature (Cooper et al., 2016; Klettke et al., 2014; Krieger, 2017; Van Ouytsel et al., 2018). According to this previous evidence indeed, older (vs. younger) adolescents, as well as LGB+ (vs. heterosexual) participants, may be more involved in sexting, and boys (vs. girls) may engage more often in nonconsensual sexting. Moreover, we tested the role of conflicts as a moderator in the relationship between the three components of love and each sexting behavior, in order to deeper understand the relational dynamics which may characterize experimental and aggravated sexting between dating partners.

In accordance with a recent and promising line of research, which aims to differentiate the determinants of experimental and aggravated sexting (Morelli et al., 2020, 2021), this study investigates sexting behaviors from three different points of view: non‐aggressive dynamic (experimental sexting), aggressive dynamic from the perpetrator perspective (nonconsensual sexting), and from the victim perspective (sexting under pressure). It is important to study sexting from different perspectives, since these different roles can frequently overlap (review meta‐analysis by Madigan et al., 2018; Mori et al., 2020), resembling the reciprocity patterns of teen dating violence (Menesini et al., 2011).

Previous research has shown that the three components of love are indicative of relationship satisfaction, involvement, and duration (Overbeek et al., 2007). So we specifically hypothesized that passion, intimacy, and commitment would be positively related to experimental sexting, which is considered by adolescents as a means to ameliorate the quality of intimate relationships (Lenhart, 2009) (H1). We conversely expected that passion, intimacy, and commitment would be negatively related to aggravated sexting, as recent studies demonstrated that dysfunctional relationship dynamics may lead to teen dating violence (Matson et al., 2021) (H2).

However, since previous studies have found limited associations between sexting and love components (Van Ouytsel et al., 2019a), and considering that aggravated sexting often occurs in a general context of dating violence (Kernsmith et al., 2018), we also hypothesized that aggravated sexting would be characterized by a conflicting relational climate as a specific risk factor, so that passion, intimacy, and commitment would predict aggravated sexting only in the presence of high perceived conflicts (H3). Previous research has demonstrated indeed that relationship conflicts are implied in different forms of teen dating aggression (Connolly et al., 2010; Connolly et al., 2010), and aggravated sexting might be considered a new kind of sexual aggression among dating partners. Moreover, recent research has demonstrated that sexting behaviors are positively related to dating conflicts (Van Ouytsel et al., 2019a), but this association has not yet been extensively explained in literature.

To our knowledge, this study is the first attempt to differentiate relational patterns associated with experimental vs. aggravated sexting, providing important implications for prevention policies and future research. Most research on adolescent sexting has been conducted in the United States. (Cooper et al., 2016; Klettke et al., 2014), and a minor part in European countries (e.g., see works by Gámez‐Guadix et al., 2017; Kopecký & Szotkowski, 2018; Van Ouytsel et al., 2017, 2018). In addition, to date very few research on sexting has been conducted in Italy (Bianchi et al., 2019; Morelli et al., 2016a, 2016b), so that the prevalence and correlates of sexting behaviors among Italian adolescents are still largely understudied.

## 3 METHOD

### 3.1 Participants and procedure

The study involved 409 middle and late adolescents (*M_age_* = 17.20, *SD_age_* = 1.61; 62.6% girls) aged from 14 to 20. Our participants were considered adolescents until 20 years old, in line with recent theories on the prolonged course of adolescence in new generations. Different authors indeed have settled the end of adolescence around 20 and 22 years old (e.g., Gentry & Campbell, 2002; Curtis, 2015; Neinstein, 2009; Steinberg, 2002).

Participants were in upper secondary schools (77%), or in the first years of university (23%). Specifically, 147 participants attended high schools (35.9%), 164 attended vocational institutes (40.1%), and 98 attended university (24%). Most of the participants (95.5%) were Italian nationals, with a small percentage (4.5%) of immigrants. Of the total sample, 31.9% reported to live in cities, 24.5% lived in suburbs, 35% lived in small towns, and 8.6% lived in rural areas. Regarding their socio‐cultural background, 20.6% of the participants’ parents were graduates or post‐graduates; 48.6% of parents had completed high school; and 30.8% had primary or middle school education levels, indicating that the reported percentages are in line with studies on representative samples of Italian adolescents (Alicandro et al., 2020). Regarding sexual orientation, 364 participants (89%) defined themselves as exclusively heterosexual, while 45 (11%) reported to be not exclusively heterosexual (LGB+).

As inclusion criteria, the participants in this study were currently in a dating relationship, or have had a dating partner in the last 12 months. A dating relationship was defined as “spending time with a person you love, like, or have a crush on” (definition adapted by Connolly et al., 2004). According to this definition, 231 adolescents (56.5%) reported to be currently involved in a dating relationship, while 178 (43.5%) have been during the past year.

Data collection was conducted via an online survey which took an average of 20 min to complete. An informed consent ensuring the voluntariness and anonymity of the research procedures was previously provided to all participants and, for school‐aged adolescents, the parents’ consent was obtained as well. The data on students attending school were collected in five public schools located in urban and suburban areas of different Italian cities, and the online survey was administered in the schools’ informatic labs under the supervision of trained psychologists. Data on students attending university were gathered with a snowball sampling method, sharing the link of the online survey on the university website. Initially, 520 participants were invited to take part in the study, and 470 of them met the inclusion criteria and accepted to participate. However, only 409 adolescents correctly completed the questionnaires, resulting in a response rate of 78.6%. This study and its procedure were approved by the Ethics Committee of Department of Social and Developmental Psychology, Sapienza University of Rome.

Power analyses have been conducted using the G*Power software program, version 3.1. Considering the conventional 80% power and 0.05 alpha significance level (Cohen, 1988), the a‐priori power analysis indicated a required sample size of 311 to detect small effect sizes (Cohen's *d* = 0.20). A post‐hoc sensitivity power analysis indicated that the actual sample size (*N* = 409) was 89% power to detect small effect sizes, and 100% power for medium (*d* = 0.50) and large (*d* = 0.80) effect sizes.

### 3.2 Measures

#### 3.2.1 Individual information

Participants reported their biological sex, age, nationality, area of residence, the grade and type of school or university they attended, and their parents’ education level. Biological sex was dummy coded as 0 (*boys*) and 1 (*girls*).

#### 3.2.2 Sexual orientation

Sexual orientation was assessed with the Kinsey scale (Kinsey et al., 1948). Participants described their sexual orientation on a 5 points Likert‐type scale, from 1 (*exclusively heterosexual*) to 5 (*exclusively homosexual*). For the purposes of our study, participants were then classified in two groups (0 = *exclusively heterosexual*; 1 = *LGB+*), following a procedure suggested in previous researches (e.g., Morelli et al., 2016b).

#### 3.2.3 Conflicts in dating relationship

The frequency of conflicts with the dating partner during a typical week was investigated with one single item (“On average, how often do you argue with your partner in a week?”). Answers were rated on a three‐point‐scale, as follows: 0 (*never, or at most once a week*); 1 (*two or three times a week*); 2 (*four or more times a week*). The item was adapted from measures of teen dating conflicts used in previous studies (Connolly et al., 2010; Furman & Buhrmester, 1992), also on Italian adolescent samples (Connolly et al., 2010).

#### 3.2.4 Components of love

The Short Triangular Love Scale (STLS; Sumter et al., 2013) was adopted to measure the individual perception of three components of love (i.e., passion, intimacy, and commitment) in the current or former dating relationship, as theorized by the Sternberg's model (1997). The instrument is composed by 12 self‐report items, with a five‐point‐response scale from 1 (*very untrue*) to 5 (*very true*), and measures the three dimensions of *passion* (four items, sample item: “I feel a strong attraction to my partner”; Cronbach's alpha of 0.78), *intimacy* (four items, sample item: “My partner and I always tell each other personal things”; Cronbach's alpha of 0.81), and *commitment* (four items, sample item: “I never want to have another partner”; Cronbach's alpha of 0.83). The good psychometric properties of this scale and its adequacy for adolescent samples have been demonstrated by Sumter et al. (2013).

For evaluating the adequacy of the STLS for Italian adolescents, two independent native‐speaking translators performed a translation of the English original items into Italian, and then the translated items were back‐translated into English. The few inconsistencies emerged by this procedure were discussed in a focus group with expert researchers in developmental psychology. This Italian version of the STLS was included in the online survey. We run a confirmatory factorial analysis (CFA) on the collected data, using the LISREL 8.80 software. Following the suggestions of previous studies (Nasser & Takahashi, 2003; Nasser & Wisenbaker, 2003), we applied an item‐parceling procedure to reduce the number of observed indicators and avoid problems of non‐convergence. In order to maximize the normality of the distribution, each STLS dimension was reduced to two parcels: the first parcel was computed as the mean score of the item with the highest negative skewness and the item with the highest positive skewness, while the second parcel was the mean score of the remaining two items. The maximum‐likelihood estimates were then computed from the sample correlation matrix. The goodness of fit of the CFA model was estimated by the relative Chi‐square test statistic (*χ* ^2^ /df), whose values are expected to range between 1 and 3 in an acceptable fit (Carmines & McIver, 1981). Moreover, we observed the following fit indexes: the comparative fit index (CFI), the normative fit index (NFI), the nonnormative fit index (NNFI), the root mean square error of approximation (RMSEA), and the standardized root mean square residual (SRMR). RMSEA values between 0.05 and 0.08 are indicative of acceptable fit (Kaplan, 2000), as are SRMR values less than 0.08, and CFI, NFI, and NNFI values greater than 0.90 (Hu & Bentler, 1999). The CFA confirmed the original three‐factors structure of the STLS in our Italian sample, χ^2^(6) = 20.12, *p* < 0.001; χ^2^/df = 3; CFI = 0.99; NFI = 0.99; NNFI = 0.98; RMSEA = 0.07; SRMR = 0.02.

#### 3.2.5 Sexting behaviors

Six items were adapted from the Sexting Behaviors Questionnaire (SBQ; Morelli et al., 2016b) in order to measure the frequency of three sexting behaviors within a dating relationship during the last year. The *sexts* were defined as “sexually suggestive or provocative text messages/images/videos shared via new technologies”, in line with previous studies (Bianchi et al., 2018; Chalfen, 2009). The first investigated dimension was *experimental sexting*, evaluating the use of sexting for improving the quality of the relationship (two items; i.e., sending sexts for attracting the attention of your partner; sending sexts for making your relationship more intriguing; Cronbach's alpha of 0.89). The two SBQ items included in this dimension are specifically framed on the definition of experimental sexting provided in literature (“youth took pictures of themselves to send to established boy‐ or girlfriends, to create romantic interest in other youth, or for attention‐seeking or other reasons that did not appear to involve elements of the Aggravated cases”, Wolak & Finkelhor, 2011, p. 3), and are also in line with experimental motives for sexting that have been described in recent studies (Bianchi et al., 2017, 2018, 2019b). The second observed dimension was *nonconsensual sexting*, composed by three items about the practice of forwarding the sexts of the partner to other people, without the partner's consent (three items; i.e., forwarding your partner's sexts via Smartphone, e.g., SMS, WhatsApp, Snapchat, without his/her consent; privately forwarding your partner's sexts via Internet, e.g., e‐mail, or social networks, without his/her consent; publicly posting your partner's sexts on social networks without his/her consent; Cronbach's alpha of 0.75). These items are in line with the description of nonconsensual sexting behaviors provided in different studies (reviews by Krieger, 2017; Madigan et al., 2018), and have been selected from the SBQ sub‐dimension “nonconsensual sexting” because they specifically refer to the partner's sexts (for a broader description of this dimension, see Morelli et al., 2021). Finally the third investigated dimension was *sexting under pressure*, evaluating the practice to engage in sexting under pressure or coercion of the partner (one item; i.e., sending sexts because your partner forced you to). Also in this case, the item was retrieved by the corresponding SBQ sub‐dimension, because it specifically refer to partner's coercion (see Morelli et al., 2021). The SBQ items were rated on a five‐point Likert‐type scale as follows: 1 (*never*); 2 (*seldom*); 3 (*sometimes or monthly*); 4 (*often or weekly*); 5 (*always or almost daily*). The SBQ sub‐dimensions have proven to be adequate in measuring sexting behaviors in adolescent samples, showing good reliabilities in different studies (Bianchi et al., 2019; Morelli et al., 2016a, 2016b). Recent research has also proven the configural invariance of SBQ dimensions across different countries (Morelli et al., 2021). Also in our study the SBQ dimensions showed good reliability values.

### 3.3 Data analysis

Data analyses were performed using the statistical program SPSS version 24.0. Descriptive statistics indicated that nonconsensual sexting and sexting under pressure were highly positively skewed and non‐normally distributed, as usually observed for other aggressive and high‐risk behaviors (Marengo et al., 2019; Wong & Raine, 2019). Thus, these variables were log‐transformed prior to perform the data analyses, in order to approximate their distribution to normality. Moreover, as expectable, 18 outliers were also detected on these two variables (Van Selst & Jolicoeur, 1994). Following a procedure suggested in recent studies (Kwak & Kim, 2017; Osborne & Overbay, 2004), the few outliers in our sample were managed with the value modification method, replacing their weights with the largest value in the sample excluding outliers. Subsequent data analyses were conducted on this adjusted sample.

Percentage frequencies of conflicts with partner, and of each sexting behavior were computed. Only for computing the sexting prevalence in our sample, participants were divided into two groups: (1) adolescents who answered 1 (*never*) to all items in each dimension (*non‐sexting group*); (2) adolescents who answered 2 to 5 (*seldom* to *always*) on at least one item in each dimension (*sexting group*). Thereafter, the continuous scores of sexting behaviors were used for the subsequent analyses. Univariate and multivariate analyses of variance (ANOVAs and MANOVAs) were run to investigate differences on the study variables according to biological sex (girls vs. boys), age (middle adolescents aged 14 to 16 vs. late adolescents aged 17 to 20), and sexual orientation (exclusively heterosexual vs. LGB+ participants). Bivariate correlations among study variables were computed. Finally, three moderated regression analyses were conducted on the continuous scores of the three sexting behaviors (experimental sexting, nonconsensual sexting, sexting under pressure).

As suggested by Aiken and West (1991), all the variables were preliminary standardized (except the dummy coded variables), and three interaction terms were computed, calculating the products of: passion × conflicts, intimacy × conflicts, and commitment × conflicts. The three moderated regression analyses were then conducted in different steps. In step 1 of each regression, biological sex, age, sexual orientation, and conflicts were entered as covariates. In step 2, the three dimensions of passion, intimacy, and commitment were entered as statistical predictors of sexting behaviors. In step 3, the three interaction terms were added to the regression equation. When significant interaction effects were found, simple slope analyses were subsequently run, by plotting the predicted values of the dependent variable as a function of the predictor, for high (1 *SD* above the mean) vs. low (1 *SD* below the mean) levels of conflicts, which was considered as the moderator (Aiken & West, 1991).

## 4 RESULTS

### 4.1 Descriptive analyses

Regarding the frequency of conflicts in dating relationships, 165 adolescents (40.3%) reported to argue with their partner never or at most once a week, 190 (46.5%) reported two or three quarrels a week, and 54 (13.2%) reported four or more quarrels a week. Regarding sexting frequencies, following the categorization described in data analyses section, 207 participants (50.6%) reported experimental sexting behaviors during the last year (vs. 202 who never engaged in experimental sexting). Nonconsensual sexting was reported by 33 participants (8.1% of the sample; vs. 376 who never engaged in this behavior). Sexting under pressure was reported by 21 participants (5.1%; vs. 388 who never reported sexting under pressure).

Regarding the ANOVA analyses conducted on the conflicts variable, no significant differences emerged for biological sex, *F*(1, 408) = 1.26, *p* = 0.26, *η^2^_partial_* = 0.00, age, *F*(1, 408) = 0.04, *p* = 0.84, *η^2^_partial_* = 0.00, and sexual orientation groups, *F*(1, 408) = 0.96, *p* = 0.33, *η^2^_partial_* = 0.00. Regarding the MANOVAs conducted on the three components of love, overall multivariate effects emerged for biological sex, *Wilk's Lambda =* 0.96, *F*(3,405) = 5.78, *p* = 0.001, *η^2^_partial_* = 0.04, and for age groups, *Wilk's Lambda =* 0.95, *F*(3,405) = 6.53, *p* < 0.001, *η^2^_partial_* = 0.05, while no difference was found for sexual orientation groups, *Wilk's Lambda =* 0.99, *F*(3,405) = 0.67, *p =* 0.57, *η^2^_partial_* = 0.00. Specifically, girls (vs. boys) reported significantly higher scores on intimacy, *F*(1,408) = 5.56, *p* = 0.02, *η^2^_partial_* = 0.01, and on commitment, *F*(1,408) = 8.73, *p* = 0.003, *η^2^_partial_* = 0.02, while late adolescents (vs. middle adolescents) reported higher scores on passion, *F*(1,408) = 18.99, *p* < 0.001, *η^2^_partial_* = 0.05, and on commitment, *F*(1,408) = 7.13, *p* = 0.008, *η^2^_partial_* = 0.02. As regards the MANOVAs performed on the continuous scores of the three sexting behaviors, significant multivariate effects were found for biological sex, *Wilk's Lambda =* 0.98, *F*(3,405) = 3.20, *p =* 0.02, *η^2^_partial_* = 0.02, and for sexual orientation groups, *Wilk's Lambda =* 0.98, *F*(3,405) = 3.49, *p =* 0.02, *η^2^_partial_* = 0.03, whereas no significant difference emerged for age, *Wilk's Lambda =* 0.99, *F*(3,405) = 0.61, *p =* 0.61, *η^2^_partial_* = 0.00. Specifically, boys (vs. girls) reported significantly higher scores in nonconsensual sexting, *F*(1,408) = 8.69, *p* = 0.003, *η^2^_partial_* = 0.02, and LGB+ participants (vs. exclusively heterosexual) reported higher scores on sexting under pressure, *F*(1,408) = 10.43, *p* = 0.001, *η^2^_partial_* = 0.03. Descriptive statistics are reported in Table 1.

**TABLE 1:** Descriptive statistics divided by gender, age, and sexual orientation groups

	Biological sex				Age				Sexual orientation				Total			
	*Girls* (*n* = 256)		*Boys* (*n* = 163)		*Middle adolescents* (*n* = 130)		*Late adolescents* (*n* = 279)		*Heterosexual* (*n* = 364)		*LGB+* (*n* = 45)					
	*M*	*SD*	*M*	*SD*	*M*	*SD*	*M*	*SD*	*M*	*SD*	*M*	*SD*	*Skew*	*Kurtosis*	*M*	*SD*
Conflicts	0.76	0.66	0.68	0.71	0.74	0.74	0.72	0.65	0.72	0.67	0.82	0.75	0.39	–0.83	0.73	0.68
Passion	4.09	0.71	4.10	0.67	3.88	0.79	4.20	0.62	4.10	0.68	4.08	0.76	–0.89	0.97	4.10	0.69
Intimacy	3.68	0.84	3.48	0.86	3.49	0.90	3.66	0.82	3.62	0.85	3.45	0.84	–0.36	–0.52	3.61	0.85
Commitment	3.59	0.99	3.29	1.03	3.28	1.12	3.57	0.95	3.49	1.02	3.40	0.97	–0.32	–0.67	3.48	1.02
Experimental sexting	1.96	1.19	1.93	1.17	2.00	1.27	1.93	1.14	1.94	1.18	2.05	1.21	1.02	–0.12	1.95	1.18
Nonconsensual sexting^a^	1.04	0.24	1.11	0.37	1.08	0.39	1.06	0.24	1.06	0.28	1.09	0.42	3.71	12.87	1.05	0.19
Sexting under pressure^a^	1.10	0.45	1.08	0.42	1.12	0.49	1.08	0.42	1.07	0.41	1.24	0.61	4.22	16.57	1.05	0.24

*Note*: ^a^ Non‐transformed mean and standard deviation values are reported for nonconsensual sexting and sexting under pressure. Then these variables were log‐transformed in data analyses in order to approximate their distributions to normality.

Correlations among study variables, as reported in Table 2, showed that conflicts were positively associated to all sexting behaviors. Regarding love components, passion showed a positive and significant correlation with experimental sexting, while intimacy showed a negative significant correlation with nonconsensual sexting. Commitment was negatively and significantly correlated to both forms of aggravated sexting (nonconsensual and under pressure).

**TABLE 2:** Bivariate Pearson's correlations on study variables and descriptive statistics for the total sample

	*1*	*2*	*3*	*4*	*5*	*6*	*7*	*8*	*9*	*10*
1. Biological sex	1									
2. Age	0.002	1								
3. Sexual orientation	0.03	–0.03	1							
4. Conflicts	0.06	–0.01	0.05	1						
5. Passion	–0.01	0.25^***^	–0.01	0.02	1					
6. Intimacy	0.12^*^	0.11^*^	–0.06	–0.02	0.57^***^	1				
7. Commitment	0.15^**^	0.14^**^	–0.03	–0.01	0.62^***^	0.63^***^	1			
8. Experimental sexting	0.01	0.01	0.03	0.13^**^	0.24^***^	0.03	0.08	1		
9. Nonconsensual sexting	–0.15^**^	0.03	–0.01	0.21^***^	–0.03	–0.17^**^	–0.16^**^	0.20^***^	1	
10. Sexting under pressure	0.02	–0.08	0.16^**^	0.11^*^	–0.02	0.03	–0.13^**^	0.27^***^	0.03	1

*Note*: ^***^ p < 0.001; ^**^ *p* < 0.01; ^*^ *p* < 0.05. Biological sex was coded as: 0 = boys; 1 = girls. Sexual orientation was coded as: 0 = heterosexual; 1 = LGB+.

### 4.2 Moderated regression analyses

The assumptions of hierarchical multiple regression analyses were preliminarily verified, with variance inflation factors falling within acceptable ranges (from 1.00 to 2.02). The first moderated regression analysis was conducted on the continuous score of experimental sexting. Step 1—in which biological sex, age, sexual orientation, and conflicts were entered as control variables—was not significant, explaining only the 1.7% of the variance. Only the frequency of conflicts with the partner was significantly and positively related with experimental sexting. In step 2—in which passion, intimacy, and commitment were entered in the model—a significant 7.5% was added to the explained variance. A significant positive effect emerged for passion, and a significant negative effect was found for intimacy. Step 3—in which the three interaction terms were entered into the regression equation—added a non‐significant 1.1% to the explained variance, and no significant interaction effects were found. The whole model explained the 10.4% of the variance in experimental sexting.

The second moderation analysis was run on the continuous log‐transformed score of nonconsensual sexting. Step 1 explained a significant 6.9% of the variance, indicating a significant negative effect for biological sex—with boys reporting higher scores than girls—and a significant positive effect for the frequency of conflicts. Step 2 added a significant 3.3% to the explained variance, detecting a significant negative effect for intimacy. Step 3 also added a significant 5.2% to the variance, and two interaction terms turned out to be significant: passion × conflicts, and commitment × conflicts. The final model explained the 15.4% of the variance in nonconsensual sexting.

The third moderation analysis investigated sexting under pressure, entered as continuous log‐transformed score. In step 1, explaining a significant 4.1% of variance, both sexual orientation and frequency of conflicts showed a significant positive relationship with sexting under pressure. Step 2 added a significant 3.9% to the explained variance, detecting a significant positive effect of intimacy and a significant negative effect of commitment. Finally, step 3 added a significant contribution of 2.0% to the explained variance, and a significant interaction effect of commitment × conflicts was found. Overall, this model explained the 10% of the variance in sexting under pressure. Statistics of the three models are reported in Table 3.

**TABLE 3:** Moderation regression analyses on three sexting behaviors

	Experimental sexting						Nonconsensual sexting						Sexting under pressure					
	Step 1		Step 2		Step 3		Step 1		Step 2		Step 3		Step 1		Step 2		Step 3	
Predictors	*B*	*SE_B_*	*B*	*SE_B_*	*B*	*SE_B_*	*B*	*SE_B_*	*B*	*SE_B_*	*B*	*SE_B_*	*B*	*SE_B_*	*B*	*SE_B_*	*B*	*SE_B_*
Biological sex	0.01	0.10	0.08	0.10	0.08	0.10	–0.32^**^	0.10	–0.25^*^	0.10	–0.23^*^	0.10	0.03	0.10	0.07	0.10	0.07	0.10
Age	0.00	0.05	–0.06	0.05	–0.06	0.05	0.03	0.05	0.04	0.05	0.04	0.05	–0.07	0.05	–0.07	0.05	–0.07	0.05
Sexual orientation	0.08	0.16	0.05	0.15	0.06	0.15	–0.03	0.15	–0.07	0.15	–0.03	0.15	0.48^**^	0.16	0.49^**^	0.15	0.53^**^	0.15
Conflicts	0.13^**^	0.05	0.12^*^	0.05	0.11^*^	0.05	0.22^***^	0.05	0.21^***^	0.05	0.21^***^	0.05	0.10^*^	0.05	0.10^*^	0.05	0.10^*^	0.05
Passion			0.36^***^	0.07	0.35^***^	0.07			0.11	0.07	0.06	0.07			0.06	0.07	0.06	0.07
Intimacy			–0.14^*^	0.07	–0.13^*^	0.07			–0.14^*^	0.06	–0.11	0.06			0.17^**^	0.06	0.18^**^	0.07
Commitment			–0.05	0.07	–0.05	0.07			–0.13	0.07	–0.12	0.07			–0.27^***^	0.07	–0.28^***^	0.07
Passion × Conflicts					0.05	0.06					0.26^***^	0.06					0.04	0.07
Intimacy × Conflicts					–0.06	0.07					–0.05	0.06					0.11	0.06
Commitment × Conflicts					–0.08	0.07					–0.22^**^	0.06					–0.19^**^	0.07
Δ*R^2^*	0.02		0.07^***^		0.01		0.07^***^		0.03^**^		0.05^***^		0.04^*^		0.04^*^		0.02^*^	
Total *R* ^2^	0.10^***^						0.15^***^						0.10^***^					

*Notes*: ^***^ p ≤ 0.001; ^**^ *p* ≤ 0.01; ^*^ *p* ≤ 0.05. Biological sex was coded as: 0 = boys; 1 = girls. Sexual orientation was coded as: 0 = heterosexual; 1 = LGB+. All variables have been standardized in advance, then unstandardized *B* regression coefficients and *B* standard errors were reported. The moderated regression analyses have been conducted on the continuous scores of each sexting behavior, including all participants (*N* = 409).

### 4.3 Slope analyses

In order to detect the direction of the significant interactions emerged in the moderation models, three simple slope analyses were run on the three relationships: (1) between passion and nonconsensual sexting; (2) between commitment and nonconsensual sexting; (3) between commitment and sexting under pressure. All the relationships were plotted for high vs. low levels of conflicts, controlling for all the variables in the models.

**FIGURE 1:**
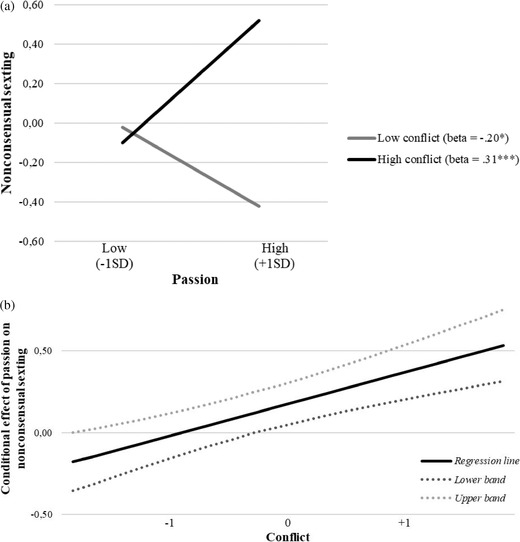
(a) Moderation effect of conflict in the relationship between passion and nonconsensual sexting. (b) Plot of confidence interval bands for conditional effect of passion on nonconsensual sexting at different levels of the moderator (conflict)

**FIGURE 2:**
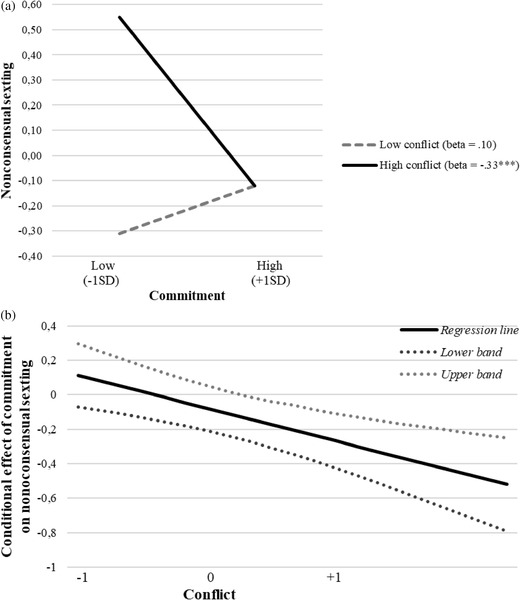
(a) Moderation effect of conflict in the relationship between commitment and nonconsensual sexting. (b) Plot of confidence interval bands for conditional effect of commitment on nonconsensual sexting at different levels of the moderator (conflict). *Note*: Dashed line represents non‐significant relationship

**FIGURE 3:**
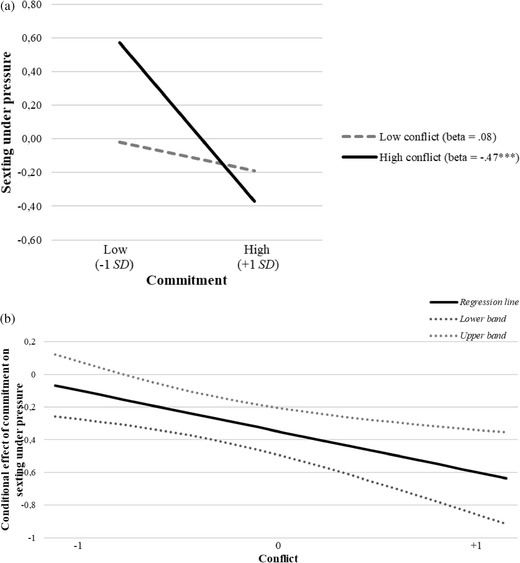
(a) Moderation effect of conflict in the relationship between commitment and sexting under pressure. (b) Plot of confidence interval bands for conditional effect of commitment on sexting under pressure at different levels of the moderator (conflict). *Note*: Dashed line represents non‐significant relationship

The first slope analysis indicated that passion was significantly and positively related to nonconsensual sexting at high levels of conflicts, *B* = 0.31, *SE_B_* = 0.08, *p* < 0.001, while conversely at low levels of conflicts the same relationship became significant and negative, *B* = ‒0.20, *SE_B_* = 0.09, *p* = 0.03 (Figure 1a and 1b). The second slope analysis showed that commitment was significantly and negatively associated to nonconsensual sexting only at high levels of conflicts, *B* = ‒0.33, *SE_B_* = 0.09, *p* < 0.001, while at low levels of conflicts, the same relationship was not significant, *B* = 0.10, *SE_B_* = 0.09, *p* = 0.29 (Figure 2a and 2b). The third slope analysis indicated that commitment was a significant negative predictor of sexting under pressure only at high levels of conflicts, *B* = ‐0.47, *SE_B_* = 0.10, *p* < 0.001. Conversely, at low levels of conflicts, commitment was not related to sexting under pressure, *B* = ‐0.08, *SE_B_* = 0.09, *p* = 0.37 (Figure 3a and 3b). Overall, high frequency of conflicts with the partner was associated with more aggravated sexting, while low frequency of conflicts appeared to be protective. In presence of high frequency of conflicts, nonconsensual sexting was predicted by high passion and by low commitment, while sexting under pressure was predicted by low commitment. However, when the frequency of conflicts was low, commitment was no more related with nonconsensual sexting and with sexting under pressure and, interestingly, high passion became inversely related to nonconsensual sexting.

## 5 DISCUSSION

In the attempt to define which relational dynamics may favor experimental and aggravated sexting behaviors between teen dating partners, the present study investigated the roles of three components of love (passion, intimacy, and commitment) and of perceived conflicts in predicting different forms of sexting (experimental, nonconsensual, and under pressure). Moreover, the interaction effects between conflicts and each component of love have been tested, providing the first evidence in literature that aggravated sexting is predicted by love components only in the presence of high perceived conflicts with the partner.

Experimental sexting, related to sharing sexts for improving the quality of dating relationship, was the most reported behavior in our sample (50.6% of participants), in line with previous studies which suggested that this is a very common practice among adolescents (Bianchi et al., 2019b; Lenhart, 2009), as spread as other offline sexual behaviors (Temple et al., 2012). Conversely aggravated sexting behaviors, either perpetrated (nonconsensual forwarding of partner's sexts) or suffered (being forced into sexting by the partner), were less reported, with percentages of 8.1% for nonconsensual sexting, and 5.1% for sexting under pressure. These results confirm previous evidence that aggravated sexting, moved by hostile intentions, is less spread among adolescents (Bianchi et al., 2018, Bianchi et al., 2019b), resembling the prevalence of other risky and aggressive online behaviors, such as problematic Internet use, meeting strangers online, and cyberbullying perpetration (Gámez‐Guadix et al., 2016).

The descriptive analyses detected sex and age‐related differences in the three components of love, confirming findings emerged in previous research (Sumter et al., 2013). Girls (vs. boys) reported higher means on intimacy in their dating relationships, in line with previous evidence (Sumter et al., 2013), and they also reported higher commitment, showing a more advanced emotional and relational development in comparison with their male peers. Late (vs. middle) adolescents reported higher passion and commitment in their dating relationships, in accordance with previous findings (Sumter et al., 2013). About sexting behaviors, results of MANOVAs were substantially in line with the individual differences emerged in the subsequent regression analyses, with boys reporting more nonconsensual sexting, and LGB+ adolescents reporting more sexting under pressure, confirming previous research (Morelli et al., 2016a, 2016b; Morelli et al., 2020; Van Ouytsel et al., 2019b).

Results of our moderation models indicated that individual differences are not present in experimental sexting, suggesting that it could be spread regardless of biological sex, age, and sexual orientation, as all other sexual behaviors between teen dating partners (Temple et al., 2012). Differently, biological sex is associated with nonconsensual sexting, as boys reported more frequent forwarding of partner's sexts in comparison with girls, confirming previous findings on this specific behavior (Morelli et al., 2016a, 2020). Sexual orientation instead is predictive only of sexting under pressure, showing that LGB+ adolescents are more frequently victims of sexting coercion by their dating partners, and confirming previous evidence about the vulnerability of sexual minority adolescents to suffer abusive forms of sexting (Van Ouytsel et al., 2019b).

Regarding the role of perceived conflicts, our moderation models clearly indicate that all three sexting behaviors are positively predicted by conflicts with partner. This evidence is consistent with recent findings on sexting in general (Galovan et al., 2018; Van Ouytsel et al., 2019a), and provides support for the association of sexting with teen dating violence (Morelli et al., 2016b). But our results also add precious information, suggesting that both experimental and aggravated forms of sexting tend to occur in presence of high conflicts between partners. We might interpret these behaviors as different strategies to manage quarrels. It is conceivable that adolescents who have frequent arguments could engage in experimental sexting as a gift to reconcile with the partner, as suggested by qualitative studies on adolescents’ motivations to sexts (Van Ouytsel et al., 2017). Otherwise, nonconsensual forwarding of the partner's sexts can be considered as a way to take revenge, threaten or punish the partner during conflicts, as suggested by studies on the revenge porn (review by Paat & Markham, 2021). Finally, consenting to sexting under pressure or coercion of the partner can constitute a way to avoid or end arguments, as suggested by some evidence on adult samples (Drouin & Tobin, 2014). During adolescence the romantic relationships are still in an explorative phase, and adolescents commonly experiment different strategies to manage first conflicts with their partners. However, these exploration may easily turn in dysfunctional dynamics, leading to more stable patterns of intimate partner violence (Wekerle & Wolf, 1999). In fact, a low conflictual relationship seems to be protective against all kinds of sexting, suggesting new directions for research and intervention on adolescent sexting.

Passion turned out to be a positive and significant predictor of experimental sexting, in line with previous findings (Van Ouytsel et al., 2019a). Our results confirm that passion plays a main role in dating relationships during adolescence, being fully developed in comparison with other components of love (Deverich, 2009; Sumter et al., 2013). Greater passion therefore seems to increase frequency of consensual sexting, as well as it can lead to other sexual behaviors with the partner. As regards nonconsensual sexting, our findings detected instead a very interesting interaction effect, indicating that passion positively predicts nonconsensual sexting only in presence of high conflicts with the partner. Conversely when the conflict is low, passion is negatively associated to the same sexting behavior. The association of high passion and high conflicts during adolescence may be interpreted as a relational climate dominated by impulsiveness and emotionality, in which violent acts may easily arise, as suggested by recent studies on the emotional context of teen dating violence (Matson et al., 2021). Moreover, since in presence of high passion consensual sexting increases, these adolescents have available sexual material about their partner, that they can easily disseminate as an intimidating or revenge act during conflicts. On the other side, when the relationship climate is not very conflictual, passion appears to be a positive resource which strengthens the emotional bond with the partner (Overbeek et al., 2007), thus contrasting the aggressive acts related to nonconsensual sexting.

Also intimacy turned out to have a very complex role. Contrary to our expectations, lower perceived intimacy was associated with more consensual sexting, perhaps enacted to improve the quality of the relationship. Lower intimacy was also associated with more nonconsensual sexting, while conversely high perceived intimacy was associated to increased vulnerability to suffer sexting coercion by the partner. Intimacy in couple relationships is a goal that is difficult to reach during adolescence, since it depends on the ongoing identity development (Erikson, 1980). Adolescent intimacy is characterized more by emotional disclosure, rather than by mature and intimate communication (Deverich, 2009). Adolescents who strive to obtain intimacy in their relationships might explore different strategies and might use consensual sexting as a way to strengthen their bond. As suggested by previous studies indeed, adolescents usually have positive expectations towards sexting, considering it a means to gain more intimacy and closeness with the partner (Lenhart, 2009). Otherwise, adolescents could also act aggressively with nonconsensual sexting, in response to a perceived weak emotional bond with their dating partner.

Conversely, high perceived intimacy with the partner, in absence of a well‐defined individual identity, may lead to risk dynamics in which the adolescents can define themselves only, or predominantly, in the context of their relationship (Duvall, 1964). In this context, the adolescents could consent to coercive sexting, as well as to other coercive dynamics, in order not to lose their partners. Studies have shown indeed that coercive sexting correlates with other forms of coercion (Kernsmith et al., 2018), and consenting to unwanted sexting is predicted by insecure attachment styles and by the desire to avoid arguments (Drouin & Tobin, 2014). Moreover, high trust and reciprocal jealousy characterize the emotional context of teen dating victimization, suggesting that strong but problematic forms of intimacy may favor vulnerability to victimization (Matson et al., 2021).

Commitment emerged to be related to aggravated sexting behaviors with a relevant protective role indicating that, in presence of high conflicts, commitment is negatively related to both nonconsensual sexting and sexting under pressure. Conversely when the conflicts are low, the same relationship is not significant. These results confirm that conflictual relational contexts favor the arising of sexting behaviors, but also suggest that in presence of high conflicts, commitment can protect against disseminating the partner's sexts online, and against suffering sexting coercion. There is evidence that low commitment in adolescent relationships is associated with more dating violence perpetration and victimization (Matson et al., 2021), and with more perceived dissatisfaction for the dating relationship (Overbeek et al., 2007). Less commitment with the partner can easily lead to destructive acts for the relationship, such as nonconsensual sexting, in the presence of high conflicts; in the same way, a low commitment could stimulate in the other partner the desire to strengthen the relationship with coercive acts, such as coercion to sext.

Commitment in adolescent dating tends to be fleeting (Deverich, 2009), since adolescents are less able to develop a stable investment in the relationship and to make long‐term planning, due to their incomplete development of prefrontal cognitive abilities (Casey et al., 2008). The incomplete prefrontal development also comports tendency to impulsive behaviors and risk taking (Casey et al., 2008). Therefore those adolescents who report higher commitment levels in their dating relationships are probably more advanced in cognitive abilities and impulse control so that, in presence of high conflicts, they can manage the relationship without neither perpetrating revenge acts, nor accepting coercive dynamics. Conversely, lower levels of commitment may be indicative of adolescents with less pre‐frontal abilities and higher impulsiveness. The act of forwarding sexual material without permission requests by itself the presence of high impulsivity and risk taking, just as it could happen for consenting to send sexts under pressure.

### 5.1 Limitations and implications

This study is the first attempt to distinguish which relational patterns could favor experimental and aggravated sexting behaviors in teen dating relationships. Our findings provide new insights into relationship dynamics that are protective or risky for nonconsensual sexting and sexting under pressure. However, the study is not exempt from some limitations. First, our results are only correlational, and no causal patterns should be inferred among the study variables. Second, we adopted online self‐report instruments, which are the most popular measurement method for sexting studies, ameliorating the participants’ perception of privacy regarding very sensitive questions. Unfortunately in self‐reports the risk for social desirability bias is high, and information about sexting behaviors could have been underreported. Third, we collected information only on biological sex, neglecting the gender identity status of participants. Moreover, the present study has not controlled for possible differences due to the actual relationship status of participants (being currently in couple vs. have been in couple in the past, but not at the moment of data collection). Possible effects related to gender identity and actual relationship status, which have not been investigated, should be further explored in future research. Finally, this study has been conducted in Italy. Our results are partially consistent with similar studies conducted in other countries (Van Ouytsel et al., 2019a) but they may represent only Italian adolescents, and may not be generalizable to different cultural contexts.

Nevertheless, our findings provide new interesting insights for research, prevention, and intervention. Future studies should investigate more in depth the role of dyadic conflicts and their interaction with love components across the lifespan in order to understand if the adolescent functioning could evolve in more violent dynamics during adulthood. The main role of conflicts as a possible trigger for aggravated sexting behaviors should be further valued in research as well as it should be taken into account in educational and prevention programs targeted to adolescents. Future studies should also consider the role of other important relational features, such as relationship duration, in predicting the frequency of different sexting behaviors. Moreover, future research should further explore the protective role of commitment in contrasting aggravated sexting, testing the possible moderation effect of impulse control, in line with the evidence on impulsivity during adolescence (Casey et al., 2008).

Educative interventions for preventing negative consequences of sexting should focus on dysfunctional relational dynamics in first dating relationships, helping adolescents to develop more effective coping strategies to manage conflicts, and discouraging the acceptance of coercive dynamics in intimate relationships. It is a common practice to undervalue the problems that adolescents may have in their dating experiences. However, our results suggest that educators and psychologists working with adolescents should consider conflicting dynamics and low commitment as important risk factors for aggravated sexting, and should sustain the development of healthy and positive models for intimate relationships.

Our results may also have implications for governmental policies about prevention of sexting incidents, suggesting the need to implement educational programs for a safe Internet use and respectful online behaviors. Our results seem to suggest that these educational programs should be enriched with elements of socio‐affective education, promoting positive models of intimate relationships and of conflicts management. These programs should be targeted to early adolescents at the age of their first dating experiences in order to prevent the occurrence of aggravated sexting behaviors within teen dating relationships. These educational interventions should be mandatory or strongly recommended in schools as well as in all other educational contexts in which preadolescents and adolescents are present. In conclusion, our study shed a new light on the different components of love which may trigger experimental and aggravated sexting behaviors, revealing the crucial moderating role of conflicting dynamics between teen dating partners. Future longitudinal research is desirable to confirm these patterns of functioning also among adult samples.

## CONFLICTS OF INTEREST

The authors declare that they have no conflict of interest.

## ETHICAL STANDARDS

All procedures performed in the study involving human participants were in accordance with the ethical standards of the institutional research committee, and with the 1964 Helsinki declaration and its later amendments or comparable ethical standards.

## INFORMED CONSENT

Informed consent was obtained from all participants included in this study.

## References

[cad20427-bib-0001] Adelman, M. , & Kil, S. H. (2007). Dating conflicts: Rethinking dating violence and youth conflict. Violence Against Women, 13(12), 1296–1318. 18046043 10.1177/1077801207310800

[cad20427-bib-0002] Aiken, L. S. , & West, S. G. (1991). Multiple regression: Testing and interpreting interactions. Thousand Oaks, CA: Sage.

[cad20427-bib-0003] Alicandro, G. , Bertuccio, P. , Sebastiani, G. , La Vecchia, C. , & Frova, L. (2020). Parental education and cancer mortality in children, adolescents, and young adults: A case‐cohort study within the 2011 Italian census cohort. Cancer, 126(21), 4753–4760. 32809229 10.1002/cncr.33146

[cad20427-bib-0081] Bianchi, D. , Morelli, M. , Baiocco, R. , & Chirumbolo, A. (2016). Psychometric properties of the Sexting Motivations Questionnaire for adolescents and young adults. Rassegna di Psicologia, 35, 5–18.

[cad20427-bib-0005] Bianchi, D. , Morelli, M. , Baiocco, R. , & Chirumbolo, A. (2017). Sexting as the mirror on the wall: Body‐esteem attribution, media models, and objectified‐body consciousness. Journal of Adolescence, 61, 164–172. 29111447 10.1016/j.adolescence.2017.10.006

[cad20427-bib-0006] Bianchi, D. , Morelli, M. , Nappa, M. R. , Baiocco, R. , & Chirumbolo, A. (2018). A bad romance: Sexting motivations and teen dating violence. Journal of Interpersonal Violence, 10.1177/0886260518817037 30537886

[cad20427-bib-0007] Bianchi, D. , Morelli, M. , Baiocco, R. , Cattelino, E. , Laghi, F. , & Chirumbolo, A. (2019a). Family functioning patterns predict teenage girls’ sexting. International Journal of Behavioral Development, 43(6), 507–514.

[cad20427-bib-0008] Bianchi, D. , Morelli, M. , Baiocco, R. , & Chirumbolo, A. (2019b). Individual differences and developmental trends in sexting motivations. Current Psychology, 10.1007/s12144-019-00398-4

[cad20427-bib-0009] Burén, J. , & Lunde, C. (2018). Sexting among adolescents: A nuanced and gendered online challenge for young people. Computers in Human Behavior, 85, 210–217.

[cad20427-bib-0010] Carmines, E. , & McIver, J. (1981). Analyzing models with unobserved variables. In G. Bohrnstedt & E. Borgatta (Eds.), Social measurement: Current issues. Beverly Hills, CA: Sage.

[cad20427-bib-0011] Casey, B. , Getz, S. , & Galvan, A. (2008). The adolescent brain. Developmental Review, 28, 62–77. 18688292 10.1016/j.dr.2007.08.003 PMC2500212

[cad20427-bib-0012] Chalfen, R. (2009). ‘It's only a picture’: Sexting,‘smutty’ snapshots and felony charges. Visual Studies, 24(3), 258–268.

[cad20427-bib-0013] Cohen, J. (1988). Statistical power analysis for the behavioral sciences (2nd ed.). Hillsdale, NJ: Erlbaum.

[cad20427-bib-0014] Connolly, J. , Craig, W. , Goldberg, A. , & Pepler, D. (2004). Mixed‐gender groups, dating, and romantic relationships in early adolescence. Journal of Research on Adolescence, 14(2), 185–207.

[cad20427-bib-0015] Connolly, J. , Friedlander, L. , Pepler, D. , Craig, W. , & Laporte, L. (2010). The ecology of adolescent dating aggression: Attitudes, relationships, media use, and socio‐demographic risk factors. Journal of Aggression, Maltreatment & Trauma, 19(5), 469–491.

[cad20427-bib-0016] Connolly, J. , Nocentini, A. , Menesini, E. , Pepler, D. , Craig, W. , & Williams, T. S. (2010). Adolescent dating aggression in Canada and Italy: A cross‐national comparison. International Journal of Behavioral Development, 34(2), 98–105.

[cad20427-bib-0017] Cooper, K. , Quayle, E. , Jonsson, L. , & Svedin, C. G. (2016). Adolescents and self‐taken sexual images: A review of the literature. Computers in Human Behavior, 55, 706–716.

[cad20427-bib-0018] Currin, J. M. , Jayne, C. N. , Hammer, T. R. , Brim, T. , & Hubach, R. D. (2016). Explicitly pressing send: Impact of sexting on relationship satisfaction. The American Journal of Family Therapy, 44(3), 143–154.

[cad20427-bib-0019] Curtis, A. C. (2015). Defining adolescence. Journal of Adolescent and Family Health, 7(2), 2–40.

[cad20427-bib-0020] Deverich, S. (2009). Love unveiled: Teenage love within the context of Sternberg's triangular theory of love. Intuition, 5, 21–25.

[cad20427-bib-0021] Drouin, M. , & Landgraff, C. (2012). Texting, sexting, attachment, and intimacy in college students' romantic relationships. Computers in Human Behavior, 28, 444.

[cad20427-bib-0022] Drouin, M. , & Tobin, E. (2014). Unwanted but consensual sexting among young adults: Relations with attachment and sexual motivations. Computers in Human Behavior, 31, 412–418.

[cad20427-bib-0023] Duvall, E. (1964). Adolescent love as a reflection of teenagers’ search for identity. Journal of Marriage and the Family, 26, 226–229.

[cad20427-bib-0024] Erikson, E. (1980). Identity and the life cycle. New York, NY: W. W. Norton and Company

[cad20427-bib-0025] Furman, W. , & Buhrmester, D. (1992). Age and sex differences in perceptions of networks of personal relationships. Child Development, 63, 103–115. 1551320 10.1111/j.1467-8624.1992.tb03599.x

[cad20427-bib-0026] Galovan, A. M. , Drouin, M. , & McDaniel, B. T. (2018). Sexting profiles in the United States and Canada: Implications for individual and relationship well‐being. Computers in Human Behavior, 79, 19–29.

[cad20427-bib-0027] Gámez‐Guadix, M. , Borrajo, E. , & Almendros, C. (2016). Risky online behaviors among adolescents: Longitudinal relations among problematic Internet use, cyberbullying perpetration, and meeting strangers online. Journal of Behavioral Addictions, 5(1), 100–107. 28092196 10.1556/2006.5.2016.013 PMC5322986

[cad20427-bib-0028] Gámez‐Guadix, M. , de Santisteban, P. , & Resett, S. (2017). Sexting among Spanish adolescents: Prevalence and personality profiles. Psicothema, 29(1), 29–34. 28126055 10.7334/psicothema2016.222

[cad20427-bib-0004] Gentry, J. H. , & Campbell, M. (2002). Developing adolescents: A reference for professionals. Washington, DC: American Psychological Association.

[cad20427-bib-0029] Graham, J. M. (2011). Measuring love in romantic relationships: A meta‐analysis. Journal of Social and Personal Relationships, 28(6), 748–771.

[cad20427-bib-0030] Hu, L. T. , & Bentler, P. M. (1999). Cutoff criteria for fit indexes in covariance structure analysis: Conventional criteria versus new alternatives. Structural Equation Modeling: A Multidisciplinary Journal, 6(1), 1–55.

[cad20427-bib-0031] Kaplan, D. (2000). Structural equation modelling. Foundations and extensions. Thousand Oaks: Sage.

[cad20427-bib-0032] Katz, J. , & Myhr, L. (2008). Perceived conflict patterns and relationship quality associated with verbal sexual coercion by male dating partners. Journal of Interpersonal Violence, 23(6), 798–814. 18272722 10.1177/0886260507313949

[cad20427-bib-0033] Kernsmith, P. D. , Victor, B. G. , & Smith‐Darden, J. P. (2018). Online, offline, and over the line: Coercive sexting among adolescent dating partners. Youth & Society, 50(7), 891–904.

[cad20427-bib-0034] Kinsey, A. C. , Pomeroy, W. B. , & Martin, C. E. (1948). Sexual behavior in the human male (pp. 157–192). Philadelphia, PA: WB Saunders Co.

[cad20427-bib-0035] Klettke, B. , Hallford, D. J. , & Mellor, D. J. (2014). Sexting prevalence and correlates: A systematic literature review. Clinical Psychology Review, 34(1), 44–53. 24370714 10.1016/j.cpr.2013.10.007

[cad20427-bib-0036] Kopecký, K. , & Szotkowski, R. (2018). Sexting in the population of children and its risks: A quantitative study. International Journal of Cyber Criminology, 12(2), 376–391.

[cad20427-bib-0037] Krieger, M. A. (2017). Unpacking “sexting”: A systematic review of nonconsensual sexting in legal, educational, and psychological literatures. Trauma Violence Abuse, 18, 593–601. 27436858 10.1177/1524838016659486

[cad20427-bib-0038] Kwak, S. K. , & Kim, J. H. (2017). Statistical data preparation: Management of missing values and outliers. Korean Journal of Anesthesiology, 70(4), 407–411. 28794835 10.4097/kjae.2017.70.4.407 PMC5548942

[cad20427-bib-0039] Lenhart, A. (2009). *Teens and sexting. How and why minor teens are sending sexually suggestive nude or nearly nude images via text messaging*. Pew Internet & American Life Project Research. Retrieved from http://ncdsv.org/images/PewInternet_TeensAndSexting_12‐2009.pdf

[cad20427-bib-0040] Levine, D. (2013). Sexting: A terrifying health risk… or the new normal for young adults?. The Journal of Adolescent Health: Official Publication of the Society for Adolescent Medicine, 52(3), 257–258. 23427782 10.1016/j.jadohealth.2013.01.003

[cad20427-bib-0041] Madey, S. F. , & Rodgers, L. (2009). The effect of attachment and Sternberg's triangular theory of love on relationship satisfaction. Individual Differences Research, 7(2), 76–84.

[cad20427-bib-0042] Madigan, S. , Ly, A. , Rash, C. L. , Van Ouytsel, J. , & Temple, J. R. (2018). Prevalence of multiple forms of sexting behavior among youth: A systematic review and meta‐analysis. JAMA Pediatrics, 172(4), 327–335. 29482215 10.1001/jamapediatrics.2017.5314 PMC5875316

[cad20427-bib-0043] Marengo, S. M. , Klibert, J. , Langhinrichsen‐Rohling, J. , Warren, J. , & Smalley, K. B. (2019). The relationship of early maladaptive schemas and anticipated risky behaviors in college students. Journal of Adult Development, 26(3), 190–200.

[cad20427-bib-0044] Matson, P. A. , Ridenour, T. A. , Chung, S. E. , Adhia, A. , Grieb, S. D. , Poole, E. , Huettner, S. , Rothman, E. F. , & Bair‐Merritt, M. H. (2021). Adolescent and young women's daily reports of emotional context and episodes of dating violence. Journal of Family Violence, 36, 271–279. 34149163 10.1007/s10896-020-00151-7 PMC8210854

[cad20427-bib-0045] McDaniel, B. T. , & Drouin, M. (2015). Sexting among married couples: Who is doing it, and are they more satisfied?. Cyberpsychology, Behavior, and Social Networking, 18(11), 628–634. 26484980 10.1089/cyber.2015.0334 PMC4642829

[cad20427-bib-0046] Menesini, E. , Nocentini, A. , Ortega‐Rivera, F. J. , Sanchez, V. , & Ortega, R. (2011). Reciprocal involvement in adolescent dating aggression: An Italian–Spanish study. European Journal of Developmental Psychology, 8(4), 437–451.

[cad20427-bib-0047] Morelli, M. , Bianchi, D. , Baiocco, R. , Pezzuti, L. , & Chirumbolo, A. (2016a). Not‐allowed sharing of sexts and dating violence from the perpetrator's perspective: The moderation role of sexism. Computers in Human Behavior, 56, 163–169.

[cad20427-bib-0048] Morelli, M. , Bianchi, D. , Baiocco, R. , Pezzuti, L. , & Chirumbolo, A. (2016b). Sexting, psychological distress and dating violence among adolescents and young adults. Psicothema, 28(2), 137–142. 27112809 10.7334/psicothema2015.193

[cad20427-bib-0049] Morelli, M. , Bianchi, D. , Cattelino, E. , Nappa, M. R. , Baiocco, R. , & Chirumbolo, A. (2017). Quando il Sexting diventa una forma di violenza? Motivazioni al sexting e dating violence nei giovani adulti. [When does sexting become a form of violence? Motivations for sexting and dating violence in young adults]. Maltrattamento e Abuso all'Infanzia, 3, 49–68.

[cad20427-bib-0050] Morelli, M. , Chirumbolo, A. , Bianchi, D. , Baiocco, R. , Cattelino, E. , Laghi, F. , Sorokowski, P. , Misiak, M. , Dziekan, M. , Hudson, H. , Marshall, A. , Nguyen, T. T. T. , Mark, L. , Kopecky, K. , Szotkowski, R. , Demirtaş, E. T. , Ouytsel, J. V. , Ponnet, K. , Walrave, M. , Zhu, T. , Chen, Y. , Zhao, N. , Liu, X. , Voiskounsky, A. , Bogacheva, N. , Ioannou, M. , Synnott, J. , Tzani‐Pepelasi, K. , Balakrishnan, V. , Okumu, M. , Small, E. , Nikolova, S. P. , & Drouin, M. (2020). The role of HEXACO personality traits in different kinds of sexting: A cross‐cultural study in 10 countries. Computers in Human Behavior, 113, 106502.

[cad20427-bib-0051] Morelli, M. , Urbini, F. , Bianchi, D. , Baiocco, R. , Cattelino, E. , Laghi, F. , Sorokowski, P. , Misiak, M. , Dziekan, M. , Hudson, H. , Marshall, A. , Nguyen, T. T. T. , Mark, L. , Kopecky, K. , Szotkowski, R. , Demirtaş, E. T. , Ouytsel, J. V. , Ponnet, K. , Walrave, M. , Zhu, T. , Chen, Y. , Zhao, N. , Liu, X. , Voiskounsky, A. , Bogacheva, N. , Ioannou, M. , Synnott, J. , Tzani‐Pepelasi, K. , Balakrishnan, V. , Okumu, M. , Small, E. , Nikolova, S. P. , Drouin, M. , Chirumbolo, A. (2021). The relationship between dark triad personality traits and sexting behaviors among adolescents and young adults across 11 countries. International Journal of Environmental Research and Public Health, 18(5), 2526. 33806314 10.3390/ijerph18052526 PMC7967332

[cad20427-bib-0080] Mori, C. , Cooke, J. E. , Temple, J. R. , Ly, A. , Lu, Y. , Anderson, N. , Rash, C. , & Madigan, S. (2020). The prevalence of sexting behaviors among emerging adults: A meta‐analysis. Archives of sexual behavior, 49, 1103–1119. 32072397 10.1007/s10508-020-01656-4

[cad20427-bib-0052] Mori, C. , Temple, J. R. , Browne, D. , & Madigan, S. (2019). Association of sexting with sexual behaviors and mental health among adolescents: A systematic review and meta‐analysis. JAMA Pediatrics, 173(8), 770–779. 31206151 10.1001/jamapediatrics.2019.1658 PMC6580450

[cad20427-bib-0053] Nasser, F. , & Takahashi, T. (2003). The effect of using item parcels on ad hoc goodness‐of‐fit indexes in confirmatory factor analysis: An example using Sarason's Reactions to Tests. Applied Measurement in Education, 16(1), 75–97.

[cad20427-bib-0054] Nasser, F. , & Wisenbaker, J. (2003). A Monte Carlo study investigating the impact of item parceling on measures of fit in confirmatory factor analysis. Educational and Psychological Measurement, 63, 729–757.

[cad20427-bib-0055] Neinstein, L. (2009). Handbook of adolescent healthcare. Philadelphia, PA: Lipppincott, Williams & Wilkins.

[cad20427-bib-0056] O'Leary, K. D. , & Smith Slep, A. M. (2003). A dyadic longitudinal model of adolescent dating aggression. Journal of Clinical Child and Adolescent Psychology, 32(3), 314–327. 12881021 10.1207/S15374424JCCP3203_01

[cad20427-bib-0057] Osborne, J. W. , & Overbay, A. (2004). The power of outliers (and why researchers should always check for them). Practical Assessment, Research, and Evaluation, 9(1), 6.

[cad20427-bib-0058] Overbeek, G. , Ha, T. , Scholte, R. , de Kemp, R. , & Engels, R. C. (2007). Brief report: Intimacy, passion, and commitment in romantic relationships—Validation of a ‘triangular love scale’ for adolescents. Journal of Adolescence, 30(3), 523–528. 17320166 10.1016/j.adolescence.2006.12.002

[cad20427-bib-0059] Paat, Y. F. , & Markham, C. (2021). Digital crime, trauma, and abuse: Internet safety and cyber risks for adolescents and emerging adults in the 21st century. Social Work in Mental Health, 19(1), 18–40, 10.1080/15332985.2020.1845281

[cad20427-bib-0060] Parker, T. S. , Blackburn, K. M. , Perry, M. S. , & Hawks, J. M. (2013). Sexting as an intervention: Relationship satisfaction and motivation considerations. The American Journal of Family Therapy, 41(1), 1–12.

[cad20427-bib-0061] Reed, L. A. , Boyer, M. P. , Meskunas, H. , Tolman, R. M. , & Ward, L. M. (2020). How do adolescents experience sexting in dating relationships? Motivations to sext and responses to sexting requests from dating partners. Children and Youth Services Review, 109, 104696.

[cad20427-bib-0062] Reed, L. A. , Tolman, R. M. , & Ward, L. M. (2016). Snooping and sexting: Digital media as a context for dating aggression and abuse among college students. Violence Against Women, 22(13), 1556–1576. 26912297 10.1177/1077801216630143

[cad20427-bib-0063] Rice, E. , Gibbs, J. , Winetrobe, H. , Rhoades, H. , Plant, A. , Montoya, J. , & Kordic, T. (2014). Sexting and sexual behavior among middle school students. Pediatrics, 134(1), e21–e28. 24982103 10.1542/peds.2013-2991

[cad20427-bib-0064] Steinberg, L. (2002). Adolescence (6th ed.). Boston, MA: McGraw Hill.

[cad20427-bib-0065] Sternberg, R. (1997). Construct validation of a triangular love scale. European Journal of Social Psychology, 27, 313–335.

[cad20427-bib-0066] Sumter, S. R. , Valkenburg, P. M. , & Peter, J. (2013). Perceptions of love across the lifespan: Differences in passion, intimacy, and commitment. International Journal of Behavioral Development, 37(5), 417–427.

[cad20427-bib-0067] Temple, J. R. , Paul, J. A. , Van Den Berg, P. , Le, V. D. , McElhany, A. , & Temple, B. W. (2012). Teen sexting and its association with sexual behaviors. Archives of Pediatrics & Adolescent Medicine, 166(9), 828–833. 22751805 10.1001/archpediatrics.2012.835 PMC3626288

[cad20427-bib-0068] Van Ouytsel, J. , Van Gool, E. , Walrave, M. , Ponnet, K. , & Peeters, E. (2017). Sexting: Adolescents’ perceptions of the applications used for, motives for, and consequences of sexting. Journal of Youth Studies, 20(4), 446–470.

[cad20427-bib-0069] Van Ouytsel, J. , Walrave, M. , Lu, Y. , Temple, J. R. , & Ponnet, K. (2018). The associations between substance use, sexual behavior, deviant behaviors and adolescents’ engagement in sexting: Does relationship context matter?. Journal of Youth and Adolescence, 47(11), 2353–2370. 30073509 10.1007/s10964-018-0903-9

[cad20427-bib-0070] Van Ouytsel, J. , Walrave, M. , & Ponnet, K. (2019a). Sexting within adolescents' romantic relationships: How is it related to perceptions of love and verbal conflict?. Computers in Human Behavior, 97, 216–221.

[cad20427-bib-0071] Van Ouytsel, J. , Walrave, M. , & Ponnet, K. (2019b). An exploratory study of sexting behaviors among heterosexual and sexual minority early adolescents. Journal of Adolescent Health, 65(5), 621–626. 10.1016/j.jadohealth.2019.06.003 31473082

[cad20427-bib-0072] Van Ouytsel, J. , Punyanunt‐Carter, N. M. , Walrave, M. , & Ponnet, K. (2020). Sexting within young adults’ dating and romantic relationships. Current Opinion in Psychology, 36, 55–59. 32480021 10.1016/j.copsyc.2020.04.007

[cad20427-bib-0073] Van Selst, M. , & Jolicoeur, P. (1994). A solution to the effect of sample size on outlier elimination. The Quarterly Journal of Experimental Psychology Section A, 47(3), 631–650.

[cad20427-bib-0074] Wekerle, C. , & Wolfe, D. A. (1999). Dating violence in mid‐adolescence: Theory, significance, and emerging prevention initiatives. Clinical Psychology Review, 19(4), 435–456. 10429844 10.1016/s0272-7358(98)00091-9

[cad20427-bib-0075] Wolak, J. , & Finkelhor, D. (2011). Sexting: A typology. Durham, NH: Crimes against Children Research Center. Retrieved from https://scholars.unh.edu/cgi/viewcontent.cgi?article=1047&context=ccrc

[cad20427-bib-0076] Wolak, J. , Finkelhor, D. , & Mitchell, K. J. (2012). *Trends in Law Enforcement Responses to Technology‐facilitated Child Sexual Exploitation Crimes: The Third National Juvenile Online Victimization Study (NJOV‐3)*. Retrieved from https://scholars.unh.edu/cgi/viewcontent.cgi?referer=https://scholar.google.it/&httpsredir=1&article=1044&context=ccrc

[cad20427-bib-0077] Wong, K. K. Y. , & Raine, A. (2019). Peer problems and low self‐esteem mediate the suspicious and non‐suspicious schizotypy—Reactive aggression relationship in children and adolescents. Journal of Youth and Adolescence, 48(11), 2241–2254. 31520236 10.1007/s10964-019-01125-9 PMC6858387

[cad20427-bib-0078] Zweig, J. M. , Dank, M. , Yahner, J. , & Lachman, P. (2013). The rate of cyber dating abuse among teens and how it relates to other forms of teen dating violence. Journal of Youth and Adolescence, 42(7), 1063–1077. 23412689 10.1007/s10964-013-9922-8

